# The Extended Statistical Analysis of Toxicity Tests Using Standardised Effect Sizes (SESs): A Comparison of Nine Published Papers

**DOI:** 10.1371/journal.pone.0112955

**Published:** 2014-11-26

**Authors:** Michael F. W. Festing

**Affiliations:** Medical Research Council Toxicology Unit, Hodgkin Building, Lancaster Road, Leicester, United Kingdom; Queen's University Belfast, United Kingdom

## Abstract

The safety of chemicals, drugs, novel foods and genetically modified crops is often tested using repeat-dose sub-acute toxicity tests in rats or mice. It is important to avoid misinterpretations of the results as these tests are used to help determine safe exposure levels in humans. Treated and control groups are compared for a range of haematological, biochemical and other biomarkers which may indicate tissue damage or other adverse effects. However, the statistical analysis and presentation of such data poses problems due to the large number of statistical tests which are involved. Often, it is not clear whether a “statistically significant” effect is real or a false positive (type I error) due to sampling variation. The author's conclusions appear to be reached somewhat subjectively by the pattern of statistical significances, discounting those which they judge to be type I errors and ignoring any biomarker where the p-value is greater than p = 0.05. However, by using standardised effect sizes (SESs) a range of graphical methods and an over-all assessment of the mean absolute response can be made. The approach is an extension, not a replacement of existing methods. It is intended to assist toxicologists and regulators in the interpretation of the results. Here, the SES analysis has been applied to data from nine published sub-acute toxicity tests in order to compare the findings with those of the author's. Line plots, box plots and bar plots show the pattern of response. Dose-response relationships are easily seen. A “bootstrap” test compares the mean absolute differences across dose groups. In four out of seven papers where the no observed adverse effect level (NOAEL) was estimated by the authors, it was set too high according to the bootstrap test, suggesting that possible toxicity is under-estimated.

## Introduction

Repeat dose toxicity tests in rodents (usually rats) are used extensively to assess the safety of drugs, chemicals, novel foods and genetically modified crops. Typically, experiments will involve a control and 1–4 dose levels or test groups following OECD guideline 408 [1]. The tests usually involve 10 animals of each sex per group or a total of 80 rats. Completely randomised designs are usual, although blocking on initial body weight is sometimes practiced.

Changes in the expression of biomarkers such as haematology, clinical biochemistry, urine composition, organ weights and sometimes others such as behavioural scores are measured to assess response. Differences between the means of the control and treated groups are usually statistically analysed, separately for each sex and biomarker, using an analysis of variance. In some cases homogeneity of variances and normality of the residuals is tested and there may be some adjustment for multiple testing using, for example, Dunnett's or other *post-hoc* tests. More rarely non-parametric test may be used. Organs are also subjected to extensive pathological assessment. The results are usually presented as tables of means and standard deviations or standard errors for each biomarker, with asterisks denoting statistical significance of differences between groups, usually at p<0.05. Interpretation depends largely on the pattern of statistical significances taking into account any observed tissue pathology.

There are several problems associate with current methods:

1. Multiple statistical testing can lead to false positives (type I errors). Although it is possible to correct for multiple tests for each biomarker using *post-hoc* tests, it is not possible to correct p-values for multiple testing of different biomarkers because it can't be assumed that biomarkers are independent. It is usually left to the investigator to decide subjectively which “statistically significant” effects are real and which are due to sampling variation (type I errors) or even technical errors.
2. Mean response across all biomarkers can't be assessed because each biomarker is measured in different units.
3. Responses which fail to reach statistical significance at p<0.05 are ignored even though they may be real effects and cumulatively they may be of toxicological importance.
4. Large tables of means and standard deviations with a few scattered asterisks are difficult to read and understand. Few graphical methods are available except for studies of growth.

These defects may lead to misinterpretation which may partially account for the relatively poor ability of these tests to predict human toxicity [2].

Multivariate statistical methods such as principle components analysis, have been used in some cases [3] in an attempt to overcome these problems, but these have not been particularly successful, and haven't replaced the traditional methods. They require access to the raw data, they are not designed for hypothesis testing, their sensitivity has not been established and as one of their aims is to reduce dimensionality, they lead to a loss of information on individual biomarkers. Toxicologists need to be able to observe the response of each biomarker to help them identify the target organ. The “false discovery rate method” has been used to deal with the problem of type I errors due to multiple testing in situations where there may be several thousand biomarkers [4]. But the method is designed to help identify those markers where there is a real response, recognising that some of them may be false. In toxicity testing the aim is not to find which biomarkers are most altered, rather it is to determine the over-all response to the test agent.

In a previous paper [5] it was suggested that many of these problems could be overcome by transforming the results to standard effect sizes (SESs). An SES is the difference between two means (say treated and control) expressed in terms of their pooled standard deviation. SESs are already widely used as a measure of response to a treatment in meta-analysis, and their use is being encouraged in other contexts [6].

The approach suggested here builds on existing methods by adding a further layer of statistical analysis using means and standard deviations, which are routinely published in papers presenting the results of toxicity tests. The advantages of this approach are:

1. It builds on, but does not replace existing methods, so should not require regulatory approval.
2. The method can be applied retrospectively, given access to tables of means and standard deviations, as access to the raw data is not required.
3. It provides a flexible range of graphical techniques to help explain the results.
4. A “bootstrap” statistical test can be used to compare the absolute mean level of response across any pair of treatment levels in all or a selected sub-set of biomarkers.
5. The hypothesis being tested using the bootstrap test becomes clearer. The null hypothesis is that there is no difference in the mean absolute response among the dose levels being compared.

The previous paper [5] used examples from a few selected papers to illustrate the graphical and statistical SES method. The aim of this paper is to compare a larger sample of papers involving repeat dose sub-acute studies, in order to illustrate the use of the SES method in a wider range of studies and make a preliminary assessment of whether the results of toxicity tests seem to be misinterpreted.

## Materials and Methods

### Data acquisition

The raw data consisted of tables of means and standard deviations (or standard errors) for each biomarker copied from the nine papers involved in this extended analysis. Papers were downloaded from PubMed Central, open access subset [21], so are all freely available.

Details of the nine papers are presented in Table 1, with the doi number (where available) being given in the list of references.

**Table 1:** Papers analysed.

Paper	First author and reference	No. Treat. Groups	Animals per group	Substance tested	No. biomarkers both sexes	Rat stock or strain	Sex	Data analysed^1^
1	Lakmichi [14]	4	8	*Corrigiola telephiifolia*	76	Wistar	Both	H,Cchem., ROWt
2	Sung [15]	4	10	n-Octane (inhaled)	41	F344	Both	Cchem
3	Matulka [18]	4	10	PolyGlycopleX	62	SD^2^	Both	H,Cchem., ROWt.
4	Johnson [19]	4	10	Resveratrol,	21	CD^3^	Both	Cchem.
5	Budin [16]	4	10	*Litsea elliptica*	25	SD	Fem only	H,Cchem,U,ROWt
6	Kim [11]	4	10	n-pentane (inhaled)	92	SD	Both	H,Cchem,ROWt
7	Seo [13]	4	10	Tetrasodium Pyrophosphate	80	SD	Both	H,Cchem,ROWt
8	Sireeratawong [17]	4	10	*Chantaleela recipe*	42	SD	Both	H,Cchem,ROWt
9	Pucaj [12]	4	10	menaquinone-7	62	SD	Both	Cchem, ROWt.

1 H = Haematology, Cchem = clinical biochemistry, U = Urine analysis, ROWt = relative organ weight.

2 Sprague-Dawley.

3 Sprague-Dawley (Charles River).

Papers were chosen largely on the basis of availability and because they had tables of means and standard deviations of measured biomarkers. Most lasted for 90 days with 10 males and 10 females per treatment group, although there are some deviations from this standard. Table 1 provides details of the compounds tested, the duration of the tests and the type and effective number of biomarkers which were measured.

Tables of means and standard deviations or standard errors in each paper were copied and pasted to Windows Notepad and asterisks and other symbols were deleted. The biomarker identity, units of measurement, gender, means and standard deviations (or standard errors) were used as column headings in an EXCEL spread-sheet. All biomarkers (haematology, clinical biochemistry, organ weights etc.) for both sexes (where used) were pooled into a single table. Some biomarkers were measured near or at the limits of detection. Where ever a standard deviation at any dose was recorded as zero or, in the few cases where there were obvious typographical mistakes, data on the biomarker were deleted. Thus the “effective number” of biomarkers was the total number in both sexes less those which were deleted. The number of biomarkers ranged from 21 to 92 (including both sexes) per dose level across all papers.

### Choice of comparisons

Three vectors of standardised effect sizes SES1, SES2 and SES3 were calculated by comparing the control group with the low, medium and high dose groups, respectively. These were used to construct the graphs and point estimates of mean absolute response at each dose level.

### Statistical methods

Standardised effect sizes (also known as Cohen's *d*) express the difference between two means in terms of their pooled standard deviations. They are widely used in meta-analysis to assess the magnitude of a response.

*Assuming equal group sizes*, which is normal in designed experiments, an SES is estimated by:
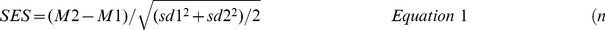
(n \ equal)Where M1 and M2 are group means and sd1 and sd2 are the corresponding standard deviations and “n” is the number in the group.

There is a direct relationship between the SES and Student's t, when group sizes are equal:
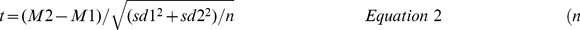
(n\ equal)

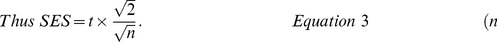
(n\ equal)Note that whereas t varies according to group size, the estimate of an SES is independent of group size although its precision depends on n.

The estimate of the SES given above is a slightly biased estimate of the population SES, depending on “n” [6]. A correction is available but has not been used here partly for simplicity, partly to retain the association with t, and partly because within an individual experiment sample size is nearly always constant, and most repeat dose toxicity experiments involve an “n” of about 10–12 animals. The correction should be applied when comparing different studies with substantially different numbers per group.

All calculations and graphics were done using the “R” statistical package [7] using R-Studio [8]. The R code for producing the plots and for the bootstrap test (see below) with a set of test data is given in Supplement S1.

### The Mean Absolute Difference (MAD) bootstrap test

Toxicity alters the level of the expression of the biomarkers. Some may go up, others may go down or be unchanged relative to the controls. The mean across all biomarkers may be zero (if the responses happen to be symmetrical), but the variation among the biomarkers expressed as SESs for a specified dose level will increase. The mean of the absolute values of SES1 (Mean(absSES1)) is a measure of the mean absolute response in the low dose group. Similarly Mean(abs(SES2)) and Mean(abs(SES3)) represent the mean absolute responses in the middle and high dose groups, respectively. In the absence of any treatment response all three means should be the same, apart from sampling variation, so the mean of the differences between any two should be zero. If the biomarkers were independent experimental units this could be tested using a one-sample t-test of the hypothesis that the mean of the absolute differences was zero. However, the biomarkers are clearly not independent observations so the parametric t-test can't be used. A “bootstrap” test is a non-parametric test which does not require the assumption of independence. Basically, it takes a random sample from the vector of differences *with replacement* (so that each number can be sampled more than once) and calculates the mean. This is repeated, say, ten thousand times to build a “bootstrap distribution” of the sampled means. The standard deviation of the bootstrap distribution can be used to estimate a 95% (or other appropriate) confidence interval for the mean difference. If this does not span zero, then the two means differ at the 5% level of probability [9].

In this case it is debatable whether a one or two tailed test is appropriate. In most cases there will be a greater response in the biomarkers at higher than at lower doses, so a one-tailed test should be used. However in some cases hormesis (a beneficial effect at low doses [10]) can't be ruled out, suggesting a two-tailed test. In this survey a two-tailed test has been used with a 5% significance level. Note that the response at the low dose level can't be tested unless there are two control groups. Differences between them would then provide an estimate of the background variation in SESs in the absence of a treatment effect. So the response at the low dose level has to be assessed in the conventional manner, looking at individual biomarkers, but these are expressed as SESs.

## Results

### Graphical presentation

**Figure 1:**
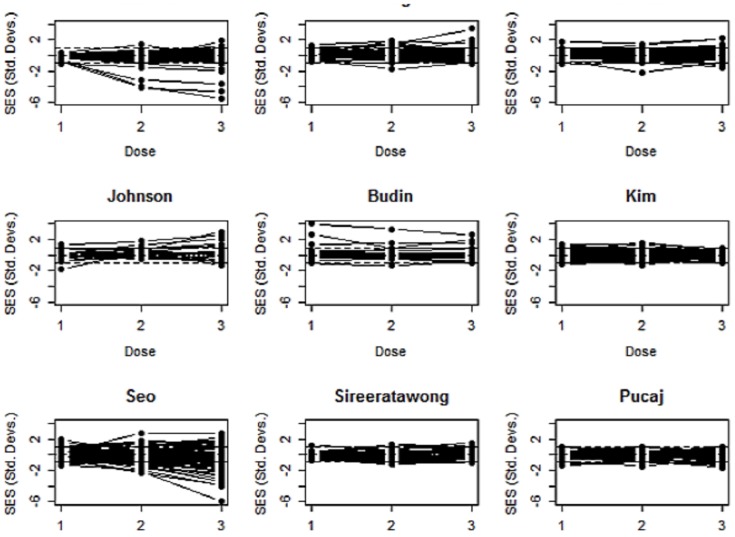
Line plots of the SESs for each biomarker, for each of the nine papers. The Y-axis scale is the same for each plot. These plots give a general impression of any dose-related effect as well as showing any outliers. Dashed lines are drawn to indicate statistical significance at p = 0.05, but at this scale they are not easy to see. There are too many biomarkers for them to be individually labelled. The response of individual biomarkers of most interest (i.e. ones that are most changed) can be seen in dotplots, an example of which is given in Fig. 4.

Line plots of SES1-SES3 for each paper are shown in Fig. 1. Patterns vary from those which seem to show no dose-response relationship such as Kim [11] or Pucaj [12]to those where many biomarkers have changed at the high dose level, such as Seo [13], or where just a few of the biomarkers have been affected, such as Lakmichi [14]. The outliers at the low dose level in the plot of the Budin [16] data are probably due to technical errors as they are more prevalent at the low than at the high dose levels, and are all associated with haematological biomarkers. There are too many biomarkers in these line plots for them to be labelled individually. However, the magnitude of the response for each biomarker can be shown in a dot-plot, discussed below.

**Figure 2:**
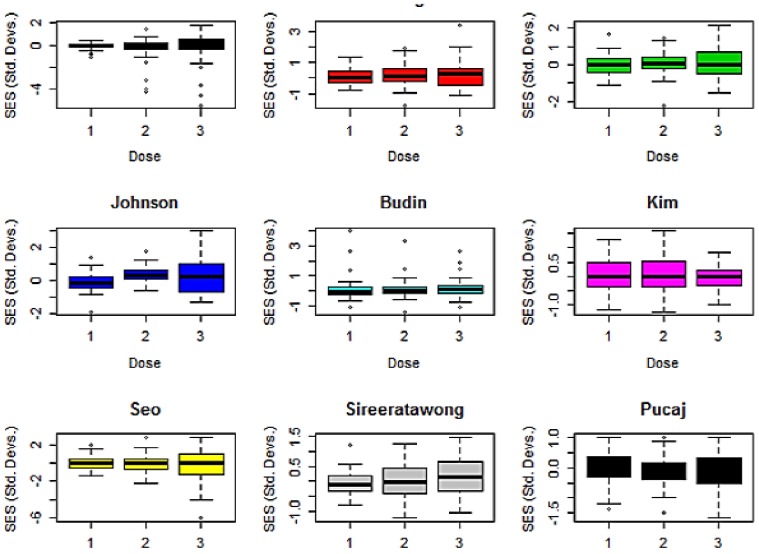
Box and whisker plots of the SESs for each of the nine papers. A treatment response shows up as a taller box and longer whiskers. The plots show outliers beyond the ends of the whiskers. Note that the Y-axis scale differs among the plots. See table 1 for details of the test compound.

**Figure 3:**
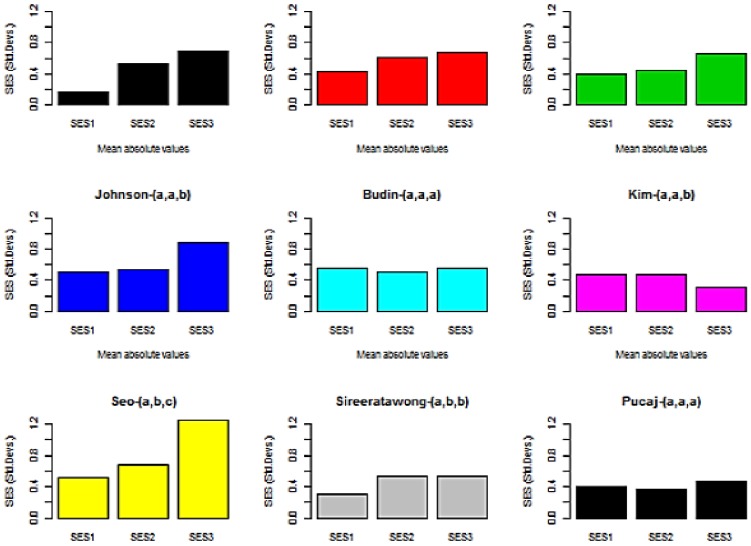
Barplots of the mean of the absolute response (Y-axis, in standard deviations) for each dose. Bars with the same letter (in the title) are not significantly different at p = 0.05 using the bootstrap test. The pattern of responses ranges from no effect (a,a,a) to a clear dose-response relationship (a,b,c).

The same data is shown as box and whisker plots in Fig. 2. These give a clearer impression of the increased variation at the higher doses in, for example, Lakmichi [14] and Sireeratawong [17], but still retain information on outliers. The mean absolute response at each dose level is shown in Fig. 3. Error bars are not used for such plots. If wanted, they would have to be estimated using a bootstrap method. However, they can't be used to compare the groups because this is paired data which is most appropriately analysed using the one-sample bootstrap test.

There is evidence of a dose-response relationship in five or six of the plots (Lakmichi [14], Sung [15], Matulka [18], Johnson [19], Seo [13] and possibly Sireeratawong [17]). Budin [16], Kim [11] and Pucaj [12] seem to not to show a dose-response relationship.

The results of the mean absolute difference (MAD) bootstrap test are shown in parenthesis in the title of each plot. Means with the same letter are not significantly different at p = 0.05. For example “Sung-(a,b,b)” means that the low and mid dose differ (p<0.05) but the mid and top do not differ at this level of probability.

### The magnitude of response detected with the bootstrap test

Averaging the response across many biomarkers would be expected to increase the power of a comparison substantially. However, the bootstrap test will depend both on sample size and the number and value of each biomarker. Further work is needed to assess the statistical power under a range of conditions, but an empirical approach provides a preliminary estimate.

Among the nine papers, each with four dose groups and three sets of SESs, there are a possible 18 bootstrap tests between adjacent dose levels. The absolute value of the 18 differences (i.e. between SES1 and SES2 or SES2 and SES3) ranged from 0.015 to.579. All nine differences of 0.16 or greater were statistically significant at p<0.05 using the bootstrap test, whereas all nine differences of less than this were not statistically significant at p = 0.05. Thus a preliminary estimate is that that these methods were able to detect as statistically significant at p<0.05, a difference in mean absolute response between two sets of SESs of about 0.16 standard deviations or more.

### Comparison with author's conclusions

According to the Environmental Protection Agency [22] the No Observable Adverse Effect Level (NOAEL) is the “..*level at which there are no statistically or biologically significant increases in the frequency or severity of adverse effects between the exposed population and its appropriate control; some effects may be produced at this level, but they are not considered as adverse, or as precursors to adverse effects.*” If a statistically significant mean absolute difference (MAD) between two sets of SESs counts as an adverse effect or a precursor to adverse effects, then it is possible to assess the level of agreement between the authors of the nine papers and the results of the SES analysis.

**Figure 4:**
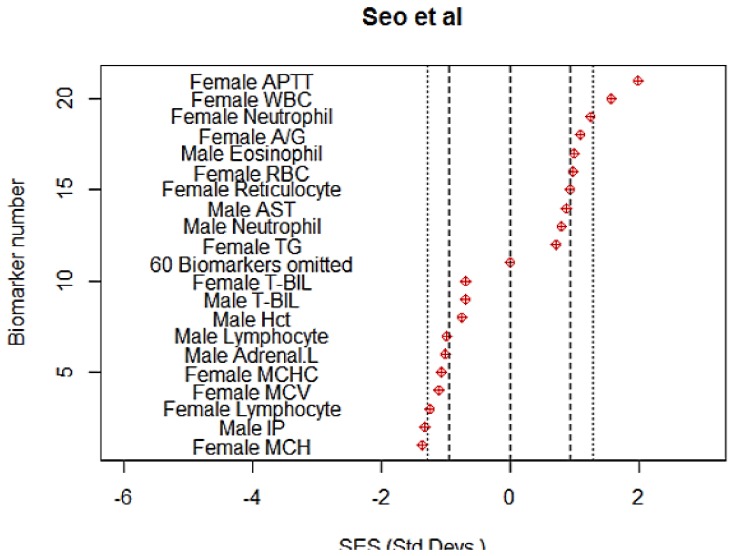
The ten most extreme responses in each direction of individual biomarkers to tetrasodium pyrophosphate at the low (250 mg/kg) dose level in the study by Seo et al. [13]. Note that 60 intermediate biomarkers were omitted and are represented by a single point at zero. Dashed lines indicate statistical significance of individual biomarkers at p = 0.05, using a t-test. Dotted lines indicate significance at p = 0.01. The authors considered this to be the NOAEL, finding no significant effects for any biomarker at this dose level. In contrast thirteen responses were found to be significant at p<0.05 in this analysis. Abbreviations are those used by the authors.

**Figure 5:**
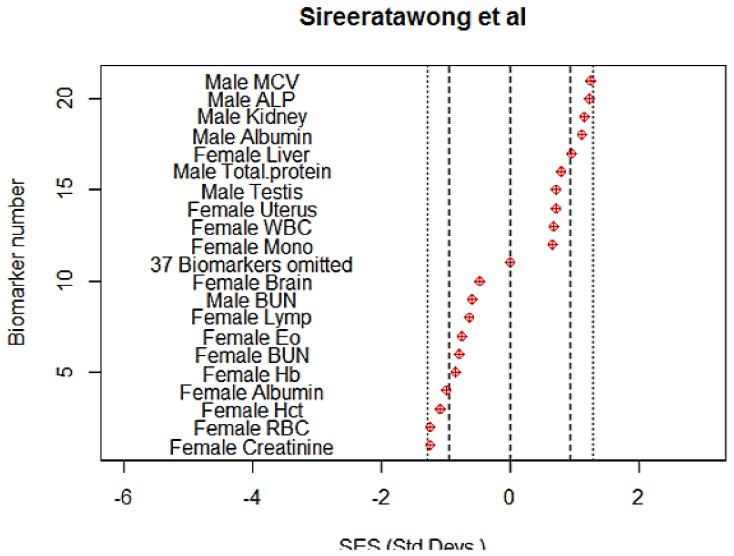
The ten most extreme individual responses in each direction to the treatment with *Chantaleela recipe* at the mid-dose (1200 mg/kg) level (Sireeratawong et al. [17]). Clearly, there is a significant response so the NOAEL should be set below this level. There was little response at the 600mg/kg dose (not shown). Abbreviations are those used by the authors.

1. **Lakmichi et al.** An aqueous extract of *Corrigiola telephiifolia Pourr.*, a Moroccan medicinal plant was administered to rats at 5, 70, and 2000 mg/kg bodyweight per day for forty days [14]. There were statistically significant (p<0.05, bootstrap test) differences in the mean absolute response between all three dose levels with indications of nephrotoxicity and hepatotoxicity. According to the authors histological examination did not reveal any treatment-related effects. The NOAEL should therefore be set at or below the lowest dose level. The authors concluded that the preparation appears safe at the doses used ethno-medicinally, but they didn't state their estimate of the NOAEL.
2. **Sung et al.** Rats were exposed to n-octane by inhalation at 0, 0.93, 2.62 and 7.48 mg/L [15]. No significant clinical or histo-pathological differences effects were observed. The authors concluded that the NOAEL was above the top level of 7.48 mg/L but that n-octane exposure should be controlled to be below the American Conference of Industrial Hygienists recommendation (300 ppm). In this SES analysis the bootstrap test found a significant difference between the low and mid dose. So, in disagreement with the authors, the NOAEL should be set at or below the low dose level.
3. **Matulka et al.** PolyGlycopleX (GPX) is a viscous dietary polysaccharide. It was administered to rats in the diet at levels of 0, 1.25, 2.5 or 5.0% for 90 days [18]. Body weight, clinical chemistry, urinalysis and haematology were measured although, surprisingly, no haematological data was presented. The authors noted a decrease in red blood cell count in the high dose males and increases in aspartate and alanine aminotransferase enzyme levels and triglycerides, while significant decreases in serum sodium, potassium and chloride concentrations were observed in the females fed 5.0% PGX. They concluded that the NOAEL was at the top dose level of 5%. The bootstrap test showed a significant (p<0.05) difference between the mid and high dose group. So this study suggests that the NOAEL should be set at the 2.5% level, one dose level lower than the NOAEL suggested by the authors.
4. **Johnson et al.** Resveratrol is a naturally occurring polyphenol with cancer preventive activity. It was administered to male and female rats at the rate of 0, 200, 400, or 1000 mg/kg/day by gavage [19]. Only growth rate, clinical biochemistry and relative organ weight data was published on the rat (a total of 21 biomarkers), although dogs were also used. According to the authors Resveratrol induced a dose-related reduction in body weight gain in female rats and a statistically significant increase in bilirubin levels at the 1000 mg/kg/day dose. The authors concluded that the NOAEL for resveratrol was 200 mg/kg/day in rats. This bootstrap test found no statistically significant difference between the 200 and 400 dose levels, although this test may have lacked power due to the low number of biomarkers, but a significant effect at the top dose level. The NOAEL according to this analysis is the 400 dose level, so Johnson et. al. were being conservative, presumably due to findings not covered by this analysis.
5. **Budin et al.** According to the authors *Litsea elliptica* Blume oil has traditionally been used to treat headache, fever, and stomach ulcer, and has also been used as an insect repellent [16]. The sub-acute toxicity was evaluated orally by gavage in female rats. Figs. 1 and 2 show that there were some large outliers at the low and middle dose levels but otherwise no evidence of a dose response relationship. The outliers are all haematological biomarkers. If all of these are omitted as technical errors, then there is no evidence of any differences between dose groups. But that is based on only 17 biomarkers. It is questionable whether there is sufficient data to reach a safe conclusion given that only one sex was used and there appear to have been technical problems with the haematology.
6. **Kim et al.** Rats were exposed by inhalation to n-pentane, a hydrocarbon solvent, for six hours per day, 5 days per week at doses of 0, 340, 1,530, and 6,885 ppm *n*-pentane [11]. Figs. 1–3 show that there was no dose related increase in the mean response in the three dose groups. The authors concluded that the NOAEL was above the top dose level. The bootstrap tests found a statistically significant *decrease* in the mean absolute response at the top dose level, presumably due to sampling effects, so it is in agreement with the authors.
7. **Seo et al.** Tetrasodium pyrophosphate (TSP) is used in processed meat products, as an emulsifier in cheese and as a preservative in soybean paste. TSP was administered by oral gavage to rats at doses of 0, 250, 500 and 1000 mg/kg five days a week for 13 weeks [13]. In the abstract the authors state that “ …in the repeated dose toxicity study, there were no significant changes in body weight in the 1,000 mg/kg treatment group, or food consumption, urinalysis, and haematology in any group”. This is misleading as 11/128 individual haemtological comparisons among groups are shown to be statistically significant at p<0.05 with six of them being significant at p<0.01 in *their* published tables. The authors considered that the NOEL was 250 and the NOAEL was 500 mg/kg/day, respectively. However, even at the low dose level of 250 mg/kg there were many individually statistically significant changes in biomarker expression (Fig. 4). The NOAEL should probably be set lower than the lowest dose level.
8. **Sireeratawong et al.** The authors state that *Chantaleela recipe*, a traditional Thai folk medicine, is composed of eight kinds of herbal plants [17]. It was fed to rats at doses of 0, 600, 1200, and 2400 mg/kg for 90 days. Haematological, clinical chemistry and absolute organ weights were measured. Histopathology showed no effect. The authors claimed that “… *Chantaleela recipe* did not cause acute or sub-chronic oral toxicities to female and male rats.” However, the bootstrap test showed a significant difference (p<0.05) in the absolute response between the low and middle dose groups, although not between the middle and top dose. Fig. 5 shows that at the middle (1200) dose there were 9/57 biomarkers which were individually statistically significant at p<0.05. Therefore the NOAEL should have been set at the low dose of 600 mg/kg.
9. **Pucaj et al.** According to the authors [12], “Menaquinone-7 (MK-7) is part of a family of vitamin K micronutrients necessary for the synthesis of blood coagulation factors and the activation of proteins involved in the building of bones and inhibition of vascular calcification.” MK-7 was administered by gavage to male and female rats at doses of 0, 2.5, 5 and 10 mg/kg body weight/day. Histopathology and biomarkers including clinical chemistry and relative organ weights were presented. The authors concluded that the NOAEL was the top dose level. The bootstrap test found no statistically significant differences between the three dose groups. These findings are in agreement with the authors with a NOAEL at 10 mg/kg/day or above.

## Discussion

Current methods for the statistical analysis of toxicity tests, with a separate analysis of each biomarker are clearly unsatisfactory for because it is impossible to distinguish between real and false positive results, effects which fail to reach statistical significance at p<0.05 are ignored and there are no satisfactory graphical method to display the results. This small survey suggests that in some cases this may be leading to a misinterpretation of the results. In four out of seven studies where the NOAEL was calculated, it was set too high according to this analysis. However, a larger sample size would be needed to assess whether under-estimation of the NOAEL is common.

The SES-based methods suggested here offer some substantial advantages. They provide an estimate of the mean absolute response, a bootstrap test to compare the response at any two dose levels, better estimates of the NOAEL, and some good graphical techniques. All this can be achieved without giving up existing methods. The extra cost are trivial as no extra experimental work is required and, as access to raw data is not necessary, the method can be applied to any set of data where means and standard deviations are available.

If these techniques were to become widely accepted, it may be worthwhile changing the design of the experiments so as to have two control groups. This would make it possible to use the bootstrap test to compare the control with the low dose group, which can't be done at present. Instead of having four doses (including the zero-dose control), two sexes and 10 animals per group (a total of 80 animals) there could be five doses (including two controls), two sexes and eight animals per group (also a total of 80 animals). The slight decrease in power from a reduced sample size would be more than compensated for by the more powerful SES statistical analysis.

It is ten years since the FDA published their “Critical path” white paper [20]. In it they stated that “The traditional tools used to assess product safety – animal toxicology and outcomes from human studies – have changed little over many decades and have largely not benefited from recent gains in scientific knowledge. The inability to better assess and predict product safety leads to failures during clinical development and, occasionally, after marketing.” Little seems to have changed since then. Maybe this method of extending the statistical analysis of the results of repeat-dose toxicity tests to minimise the chance of misinterpretation could represent one small step in reducing the cost of drug development.

## Conclusions

The chances of misinterpreting the results of repeat-dose toxicity tests can be reduced by extending the statistical analysis using standardised effect sizes. The methods provide for good graphical presentation and an over-all test of mean absolute response. These methods are recommended as an extension to the statistical analysis of all toxicity studies where multiple quantitative biomarkers are measured.

## Supporting Information

Supplement S1 **Supporting information.** R code and test data.
(DOCX)
