# Supplementary material for: The Extended Statistical Analysis of Toxicity Tests Using Standardised Effect Sizes (SESs): A Comparison of Nine Published Papers
Source: PLoS One. 2014 Nov 26;9(11):e112955. doi: 10.1371/journal.pone.0112955 (PMC4245118; doi:10.1371/journal.pone.0112955)
Supplement: Supplement S1 — Supporting information. R code and test data. (DOCX) [file pone.0112955.s001.docx]

S1. Supporting information. R code and test data.

All the plots and the bootstrap test were done using the “R” statistical package [7] run using “R Studio”[8]. Both of these programs can be downloaded free of charge.

The R code suitable for producing individual plots and the bootstrap test is given below. It is written as simply as possible, but a reasonable knowledge of R is required to run it because it requires manual intervention in several places. For example “n” has to be entered manually. It is also necessary to make adjustments if the data is ± standard errors instead of standard deviations, and the X and Y limits of the plots will need to be adjusted to ensure that they fit the data. The dotplots and bootstrap test require a decision on which dose levels are to be plotted or compared. Any code or text following a hash mark # is ignored by R so is used to give instructions. A small set of test data is also given to show the required format of data and headings accepted by the code. This can be used for practice. The columns can be in any order, but column names should be as shown and are case sensitive. Beware that authors sometimes claim that their data is ± SD when it is in fact ± SE. Users are free to alter the code as they wish.

Please note that **anyone using this code does so at their own risk**. The author accepts no responsibility for any errors or omissions or any consequential losses arising from its use.

| Table S1. Data for practice in use of the R code (A subset of data taken from Seo [13]. This data should be transferred to a spread-sheet such as EXCEL and copied to the clipboard in order to be read into the R software program using the R code given in here. Note that each cell in EXCEL must only have a single word or number. If two words are required there must be no space between them.. | | | | | | | | | | |
| --- | --- | --- | --- | --- | --- | --- | --- | --- | --- | --- |
| Sex | Biomarker | Units | X1 | sd1 | X2 | sd2 | X3 | sd3 | X4 | sd4 |
| Male | WBC | (K) | 7.2 | 1.83 | 6.62 | 0.71 | 7.76 | 1.58 | 9.35 | 1.31 |
| Male | Neutrophil | (%) | 14.2 | 4.6 | 18.6 | 6.2 | 20.1 | 6.6 | 24.8 | 4.9 |
| Male | Lymphocyte | (%) | 80.1 | 4.5 | 74.8 | 6.2 | 73.6 | 6.9 | 68.7 | 6 |
| Male | Monocyte | (%) | 3.6 | 1.1 | 3.9 | 1.6 | 3.4 | 1.1 | 4.1 | 1.8 |
| Male | Eosinophil | (%) | 1.4 | 0.5 | 1.9 | 0.5 | 2.3 | 0.7 | 1.8 | 0.6 |
| Male | RBC | (M) | 8.56 | 0.19 | 8.43 | 0.34 | 8.55 | 0.42 | 8.19 | 0.19 |
| Male | Hb | (g/dl) | 15.3 | 0.5 | 14.9 | 0.7 | 14.8 | 0.8 | 14.4 | 0.3 |
| Male | Hct | (%) | 45.5 | 1.4 | 44.3 | 1.8 | 44.4 | 2.2 | 43.1 | 0.8 |
| Male | MCV | (fL) | 53.1 | 1.1 | 52.5 | 1.3 | 52 | 2.7 | 52.7 | 0.9 |
| Male | MCH | (pg) | 17.9 | 0.4 | 17.6 | 0.5 | 17.3 | 1 | 17.6 | 0.4 |
| Male | MCHC | (g/dL) | 33.8 | 0.5 | 33.5 | 0.5 | 33.2 | 0.6 | 33.4 | 0.5 |
| Male | Reticulocyte | (%) | 2.04 | 0.34 | 2.1 | 0.3 | 2.38 | 0.52 | 2.37 | 0.38 |
| Male | PLT | (K) | 1148.2 | 124.4 | 1071.6 | 98.4 | 1213.9 | 123.4 | 1264.5 | 110.5 |
| Male | PT | (sec) | 17.7 | 0.5 | 17.6 | 0.6 | 16.7 | 0.6 | 16.5 | 0.6 |
| Male | APTT | (sec) | 20.3 | 1.2 | 19.5 | 1.9 | 19.4 | 1.2 | 18.4 | 1.1 |
| Female | WBC | (K) | 3.72 | 1.86 | 6.84 | 2.09 | 4.44 | 0.66 | 6.39 | 1.83 |
| Female | Neutrophil | (%) | 12.2 | 3.7 | 17.7 | 5 | 17.3 | 7.3 | 20.9 | 6.3 |
| Female | Lymphocyte | (%) | 81.4 | 5 | 74.4 | 6.1 | 75.4 | 7.4 | 73.2 | 7 |
| Female | Monocyte | (%) | 3.6 | 1.5 | 3.7 | 0.2 | 3.8 | 1.7 | 3.3 | 0.9 |
| Female | Eosinophil | (%) | 2.1 | 0.9 | 1.9 | 0.3 | 2.6 | 1.1 | 2.1 | 0.6 |
| Female | RBC | (M) | 7.82 | 0.28 | 8.16 | 0.4 | 7.96 | 0.23 | 7.82 | 0.25 |
| Female | Hb | (g/dl) | 14.4 | 0.5 | 14.5 | 0.6 | 14.6 | 0.4 | 14.1 | 0.4 |
| Female | Hct | (%) | 42.3 | 1.5 | 43.1 | 2 | 42.8 | 1.3 | 42 | 1.2 |
| Female | MCV | (fL) | 54.1 | 1.2 | 52 | 2.4 | 53.8 | 1.3 | 53.7 | 0.9 |
| Female | MCH | (pg) | 18.5 | 0.5 | 17.5 | 0.9 | 18.3 | 0.5 | 18.1 | 0.2 |
| Female | MCHC | (g/dL) | 34.1 | 0.4 | 33 | 1.4 | 34.1 | 0.2 | 33.7 | 0.4 |
| Female | Reticulocyte | (%) | 1.79 | 0.41 | 2.11 | 0.26 | 2.02 | 0.49 | 2.27 | 0.42 |
| Female | PLT | (K) | 1153.7 | 171.4 | 1153.2 | 105.1 | 1169.5 | 427.3 | 1441.4 | 210.2 |
| Female | PT | (sec) | 16.9 | 0.8 | 17.1 | 0.6 | 17.1 | 1 | 16.4 | 0.9 |
| Female | APTT | (sec) | 16.4 | 0.8 | 18.8 | 1.5 | 16.5 | 0.9 | 16.7 | 0.8 |
| Male | TP | (g/dL) | 6.2 | 0.4 | 6 | 0.2 | 5.4 | 0.3 | 4.8 | 0.3 |
| Male | Albumin | (g/dL) | 2.5 | 0.2 | 2.4 | 0.1 | 2.3 | 0.1 | 2.1 | 0.1 |
| Male | A/G | Ratio | 0.67 | 0.05 | 0.68 | 0.06 | 0.73 | 0.02 | 0.74 | 0.05 |
| Male | T-BIL | (mg/dL) | 0.078 | 0.01 | 0.071 | 0.01 | 0.074 | 0.015 | 0.076 | 0.031 |
| Male | ALP | (U/L) | 270 | 49 | 278 | 36 | 277 | 74 | 227 | 55 |
| Male | AST | (U/L) | 99 | 21 | 117 | 20 | 103 | 16 | 138 | 25 |
| Male | ALT | (U/L) | 35 | 5 | 37 | 10 | 31 | 4 | 39 | 13 |
| Male | BUN | (mg/dL) | 11.5 | 1.4 | 11.5 | 2.2 | 12.2 | 1.3 | 13.6 | 1.7 |

R code to draw the plots and do the bootstrap test.

It is assumed that the user has some familiarity with R and RStudio and can make changes to the code where required. This code can be copied directly into the code section in RStudio. The data in Appendix 1 can be used to test it. Sections of the code are marked and run as seems appropriate. Some manual intervention is required, as described in many cases following a hash mark (R ignores anything on a line after #).

This code is used at your own risk. The author accepts no responsibility for any inaccuracies/mistakes and any consequential losses.

#######################################################################

### test code in paper 2.

#######################################################################

#####Calulates ses1,ses2,ses3 and makes suitable plots and a bootstrap test

## The code should be worked through sequentially running a few lines at a time

## Some knowledge of R is required. Code can be adapted for other comparisons

#######################################################################

##### Calulates ses1,ses2,ses3 and makes suitable plots and a bootstrap test

##### The code should be worked through sequentially running a few lines at a time

rm(list=ls(all=TRUE))

rawdata<-read.table("clipboard", header=T) ## Data should have been copied to the clipboard

attach(rawdata)

names(rawdata) # A check to make sure the data is present

n<-10 #Sample size. Change if necessary

nchar<-length(X1)

Title<-"Test data" # change title as required

#sd1<-sd1*sqrt(n) # unblock if SEM not SDs

#sd2<-sd2*sqrt(n) # Unblock if SEM not SDs

#sd3<-sd3*sqrt(n) # unblock if SEM not SDs

#sd4<-sd4*sqrt(n) # unblock if SEM not SDs

#################################

ses1<-(X2-X1)/sqrt((sd1^2+sd2^2)/2)

ses2<-(X3-X1)/sqrt((sd1^2+sd3^2)/2)

ses3<-(X4-X1)/sqrt((sd1^2+sd4^2)/2)

ses.all<-c(ses1,ses2,ses3)

L05<-qt(.975,2*n-2)*(sqrt(2)/sqrt(n)) # Line for 5% significance

L01<-qt(.005,2*n-2)*(sqrt(2)/sqrt(n)) # Line for 1% significance

################################## mean absolute responses

mean(abs(ses1))

mean(abs(ses2))

mean(abs(ses3))

################################# Dotplot

############## may need to adjust xlim

SES<-ses1 ###### Choose which ses to plot

Dose <- "Low dose" ###### Change label appropriately

Nomit<-nchar-20

X<-(1:21)

o<-order(SES)

Z1<-SES[o]

Z2<-c(Z1[1:10],0,Z1[(nchar-9):nchar])

par(cex=1)

#############may need to change title, xlim and sub

plot(X~Z2, ylab="Biomarker number", xlab="SES (Std. Devs.) ",

xlim=c(-6,3), pch=10, col="red", main=Title, sub=Dose)

abline(v=0, lty=2)

abline(v=-L05, lty=2)

abline(v=L05, lty=2)

abline(v=L01, lty=3)

abline(v=-L01, lty=3)

ID<-paste(Sex[o], Biomarker[o])

ID1<-ID[1:10]

ID2<-ID[(nchar-9):nchar]

ID4<-paste(Nomit, "Biomarkers omitted")

ID3<-c(ID1,ID4, ID2)

par(cex=.6)

text(-4,X,ID3) #### May need to change X range

##################################### Boxplot

############## May want to change colours

par(cex=1)

D<-gl(3,nchar,3*nchar)

boxplot(ses.all~D, xlab="Dose", ylab="SES (Std. Devs.)",

main=Title, col=c(3,"yellow",2))

abline(h=0, lty=2)

#################################### Lineplot (stripchart)

############## May need to alter ylim

d1<-rep(1, nchar)

d2<-rep(2,nchar)

d3<-rep(3,nchar)

i=1

C<-c(ses1[i],ses2[i], ses3[i])

D<-c(d1[i],d2[i],d3[i])

stripchart(C~D,vert=T, xlab="Dose 1 to 3", ylim=c(-6,4),

ylab="SES (Std. Devs.) ", main=Title)

for(i in 1:nchar){

C<-c(ses1[i],ses2[i], ses3[i])

lines(C~D,type="b", pch=16)

i=i+1}

abline(h=L05, lty=2)

abline(h=-L05,lty=2)

abline(h=0, lty=3)

####################################### Barplot

############## Change colours if wanted

A<-mean(abs(ses1))

B<-mean(abs(ses2))

C<-mean(abs(ses3))

all.means=c(A,B,C)

barplot(all.means,main=Title,sub="Author, a, b, c", ylim=c(0,1.5),

ylab="SES (Std.Devs.)", xlab="Dose", col=c("green","yellow","red"), names=c(1,2,3))

###################################### Bootstrap test for abs. mean response

################# Choose which means (ses) to compare

X<-abs(ses2)-abs(ses1)

result=0

N=10^4

result=numeric(N)

for(i in 1:N){

z=sample(X,nchar, replace=T)

result[i]=mean(z)}

quantile(result, c(0.025,0.975)) ###gives the 95% confidence interval

mean(X)

hist(X, col="yellow", main="Histogram of differences")

length(X)

###################################### A table of SESs if wanted.

Table<-cbind(paste(Biomarker,Sex,Units),round(ses1,digits=2),round(ses2,digits=2),

round(ses3, digits=2))

Table

#############################################################
